# Quercetin Attenuates Quinocetone-Induced Cell Apoptosis In Vitro by Activating the P38/Nrf2/HO-1 Pathway and Inhibiting the ROS/Mitochondrial Apoptotic Pathway

**DOI:** 10.3390/antiox11081498

**Published:** 2022-07-30

**Authors:** Chongshan Dai, Qinzhi Zhang, Linjie Shen, Gaurav Sharma, Haiyang Jiang, Zhanhui Wang, Jianzhong Shen

**Affiliations:** 1 College of Veterinary Medicine, China Agricultural University, No.2 Yuanmingyuan West Road, Beijing 100193, China; 2016305010216@cau.edu.cn (Q.Z.); slj@cau.edu.cn (L.S.); haiyang@cau.edu.cn (H.J.); wangzhanhui@cau.edu.cn (Z.W.); sjz@cau.edu.cn (J.S.); 2 Beijing Key Laboratory of Detection Technology for Animal-Derived Food Safety, Beijing 100193, China; 3 Advanced Imaging Research Center, University of Texas Southwestern Medical Center, Dallas, TX 75390, USA; Gaurav.sharma@utsouthwestern.edu

**Keywords:** quercetin, quinocetone, oxidative stress, apoptosis, p38/Nrf2/HO-1 pathway, NF-κB pathway

## Abstract

Quinocetone (QCT), a member of the quinoxaline 1,4-di-*N*-oxides (QdNOs) family, can cause genotoxicity and hepatotoxicity, however, the precise molecular mechanisms of QCT are unclear. This present study investigated the protective effect of quercetin on QCT-induced cytotoxicity and the underlying molecular mechanisms in human L02 and HepG2 cells. The results showed that quercetin treatment (at 7.5–30 μM) significantly improved QCT-induced cytotoxicity and oxidative damage in human L02 and HepG2 cells. Meanwhile, quercetin treatment at 30 μM significantly inhibited QCT-induced loss of mitochondrial membrane potential, an increase in the expression of the CytC protein and the Bax/Bcl-2 ratio, and an increase in caspases-9 and -3 activity, and finally improved cell apoptosis. Quercetin pretreatment promoted the expression of the phosphorylation of p38, Nrf2, and HO-1 proteins. Pharmacological inhibition of p38 significantly inhibited quercetin-mediated activation of the Nrf2/HO-1 pathway. Consistently, pharmacological inhibitions of the Nrf2 or p38 pathways both promoted QCT-induced cytotoxicity and partly abolished the protective effects of quercetin. In conclusion, for the first time, our results reveal that quercetin could improve QCT-induced cytotoxicity and apoptosis by activating the p38/Nrf2/HO-1 pathway and inhibiting the ROS/mitochondrial apoptotic pathway. Our study highlights that quercetin may be a promising candidate for preventing QdNOs-induced cytotoxicity in humans or animals.

## 1. Introduction

**Figure 1:**
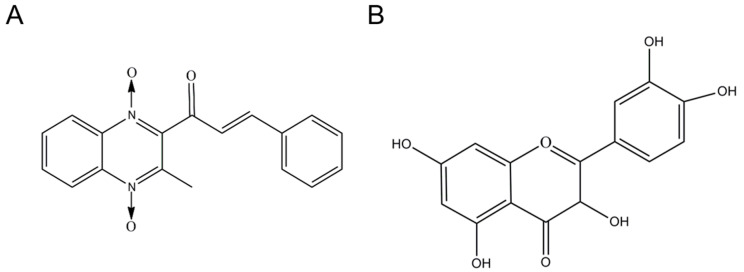
The structure of quinocetone (QCT; (**A**)) and quercetin (**B**).

Quinoxaline-1,4-dioxides (QdNOs), a class of quinoxaline derivatives, have been widely employed in agricultural and medicinal fields across the world due to their antibacterial, antitubercular, anticandidal, antiprotozoal, and anticancer activity [1,2,3,4]. Quinocetone (QCT) (i.e., 3- methyl-2-quinoxalin benzenevinylketo-1, 4-dioxide, Figure 1A), a relatively new member of QdNOs family, was first approved as an antibacterial agent in China since 2003 [5]. Animal experimental investigations revealed that oral exposure of QCT is rarely absorbed in rats, pigs, broilers, and carp, and exhibited low toxic effects [6,7]. The LD_50_ doses (i.e., median lethal dose) of QCT in rats and mice were about 8687.3 mg/kg body weight and 15,848.9 mg/kg body weight, respectively [8]. In contrast, QCT exhibited potent cytotoxic effects on a variety of cell lines, including human L02 cells, HepG2 cells, primary porcine adrenocortical cells, Vero cells, human adrenocortical cells (i.e., NCI-H295R cells), and human primary peripheral lymphocytes [5,9,10,11,12,13]. For example, Yang et al. found that QCT treatment at a lower dose (i.e., at 2.5 μg/mL for 4 h) might cause significant cytotoxicity and genotoxicity in human peripheral lymphocytes [12]. In addition, the chronic oral administration of QCT in rats or mice could result in marked genotoxicity, hepatotoxicity, nephrotoxicity, and immunotoxicity [8,11,14,15]. Therefore, understanding the precise molecular mechanisms of QCT is required for the development of cytoprotective agents, risk assessment, and combination therapy of QCT in clinical practice.

Previous research has shown that the generation of reactive oxygen species (ROS), oxidative stress, apoptosis, mitochondrial malfunction, autophagy, and the inflammatory response are all plausible molecular pathways of QCT-induced toxicity [10,16,17,18,19,20,21]. Several pathways have been identified, including NF-E2-related transcription factor 2 (Nrf2)/heme oxygenase-1 (HO-1), nuclear factor-κB (NF-κB), activating transcription factor 6 (ATF6)/death-associated protein kinase (DAPK1) pathway, tumor necrosis factor-α (TNF-α), phosphoinositide-3-kinase (PI3K)/protein kinase B (Akt), Wnt/β-catenin, and mitogen-activated protein kinase (MAPK) pathways [10,17,18,19,20,22,23,24]. However, the precise mechanisms of QCT remain largely unknown, resulting in a lack of effective protective medications.

Quercetin (3,3′,4′,5,7-pentahydroxyflavone; Figure 1B), a natural flavonoid compound, could be found and isolated in various fruits, vegetables, tea, red wine, and various medicinal herbs [25]. Quercetin was shown to have anti-oxidative stress, anti-inflammatory, anti-microbial, neuroprotection, anti-cancer, and immunological regulatory properties in multiple previous investigations [26,27]. Additionally, the recent study suggests that quercetin may be useful in the prevention and treatment of SARS-CoV-2-related disease. [28]. Moreover, several studies found that quercetin supplementation could significantly reduce hepatotoxicity and nephrotoxicity caused by antibacterial drugs (e.g., rifampicin and ciprofloxacin) or environmental toxins (e.g., copper, nickel, ochratoxin A, and benzo(a)pyrene) by inhibiting oxidative stress and the inflammatory response [29,30,31,32,33]. Currently, there is limited information on whether quercetin could provide the protective effects on QCT-induced cytotoxicity. Therefore, in the present study, the protective effects of quercetin on QCT-induced cytotoxicity and the underlying molecular mechanisms using in vitro cell models were investigated.

## 2. Materials and Methods

### 2.1. Chemicals and Reagents

QCT (C18Hl4N203, CAS NO.81810-66-4; purity ≥ 98%) was purchased from Zhongmu Pharmaceutical Co. Ltd. (Wuhan, China). Quercetin (purity ≥ 98%) was purchased from Aladdin Reagent Co., Ltd. (Shanghai, China). Nrf2 inhibitor brusatol was purchased from Tauto Biotech. (Shanghai, China). Dulbecco’s Modified Eagle’s Medium (DMEM) and fetal bovine serum (FBS) were purchased from Invitrogen (Gibco, Grand Island, NY, USA). P38 inhibitor SB203580 and phenylmethanesulfonylfluoride (PMSF) were purchased from Beyotime (Haimen, China). All other reagents were the analytical-reagent-grade.

### 2.2. Cell Culture

Human L02 (RRID: CVCL_6926; STR analysis is shown in Suppl. Table S1) and HepG2 cells were purchased from Shanghai Cell Bank of Chinese Academy of Sciences. The detailed culture conditions have been well described in our previous study [34].

### 2.3. Measurement of Cell Viability

To study the protective effect of quercetin supplementation on QCT-induced cytotoxicity, cell viabilities were measured according to methods described in our previous study [35]. Briefly, L02 cells at a density of 1 × 10^4^ cells/well were seeded in a 96-well plate. After culturing for 16 h, the cells were pre-treated with quercetin at final concentrations of 7.5, 15, and 30 μM for 2 h, then co-treated with QCT at a final concentration of 5 μg/mL for an additional 24 h. In the control group, L02 cells were treated with 0.2% DMSO as the vehicle control. After treatment, the cells were cultured with a fresh medium containing 10 μL CCK-8 solution and incubated for 1 h at 37 °C. Then, the value of absorbance at 450 nm was read using a microplate reader (Tecan Trading AG, Männedorf, Switzerland). The final values were normalized to the control group. Three independent experiments were performed.

Similarly, HepG2 cells were pretreated with quercetin at concentrations of 7.5, 15, and 30 μM, followed by co-treatment with QCT at 10 μg/mL for an additional 24 h. The dose of QCT followed the methods described in our previous study [17]. After treatment, cell viabilities were measured using the CCK-8 method.

### 2.4. Measurement of the Levels of AST and ALT

L02 cells at a density of 2 × 10^5^ cells/well were seeded into a 12-well plate. After 16 h, the cells were pretreated with quercetin at concentrations of 7.5, 15, and 30 μM for 2 h, then treated with or without QCT at a final concentration of 5 μg/mL for an additional 24 h. Then, the culture mediums were centrifuged for 15 min at 3000× *g*, and the dead cells and debris were removed. The supernatants were collected. The levels of aspartate transferase (AST) and alanine transaminase (ALT) were measured using an automatic biochemical analyzer (model 7600; Hitachi Ltd., Tokyo, Japan).

### 2.5. Measurement of Cell Apoptosis

Cell apoptosis was analyzed using the Hoechst 33342 staining method (Beyotime, Shanghai, China) according to methods described in our previous study [10]. In brief, L02 cells at a density of 2 × 10^5^ cells/well were plated into a 12-well plate and pretreated with quercetin at concentrations of 7.5, 15, and 30 μM for 2 h, followed by treatment with or without QCT at 5 μg/mL for an additional 24 h. After treatment, the cells were stained for 30 min with 1 μg/mL Hoechst 33342 in the dark. Then, nuclear morphological changes were observed using a fluorescence microscope at 340 nm of excitation wavelength and 460 nm of emission wavelength) (Leica Microsystems, Wetzlar, Germany). Apoptotic cells were defined as those with increased chromatin condensation and DNA fragmentation.

To further confirm the protective effect of quercetin on QCT-induced apoptosis, HepG2 cells were treated with quercetin at concentrations of 7.5, 15, and 30 μM for 2 h, followed by treatment with or without QCT at 10 μg/mL for an additional 24 h. Cell apoptosis rates were measured using the Hoechst 33342 staining method mentioned above.

### 2.6. Measurement of DNA Damage

DNA damage was assessed by the comet assay according to methods described in the previous study [34,36]. In brief, L02 cells at a density of 2 × 10^5^ cells/well were plated into a 12-well plate and pretreated with quercetin at 7.5, 15, and 30 μM for 2 h, followed by treatment with or without QCT at 5 μg/mL for additional 4 h. In all groups, cell viabilities were more than > 80%. Comet assay was performed using an Oxiselect Comet Assay^®^kit (Cell Biolabs, San Diego, CA, USA) according to the manufacturer’s instructions. After staining with Vista Green DNA dye for 10 min, the changes of the comet were observed using fluorescence microscopy (excitation wavelength: 488 nm; emission wavelength: 525 nm) (Leica Microsystems, Wetzlar, Germany). A total of 100 cells per sample were analyzed by Comet Assay Software Project (CASP) 1.2.2 (University of Wroclaw, Wroclaw, Poland). The percent (%) of tail DNA is calculated as below:

% of tail DNA = (% of tail DNA intensity/cell DNA intensity) × 100%

### 2.7. Measurement of Intracellular ROS Generation, Malondialdehyde (MDA), Superoxide Dismutase (SOD), Catalase (CAT), and Reduced Glutathione (GSH) Levels

The levels of intracellular ROS were measured using ROS-specific fluorescent dye 2,7-dichlorofluorescein diacetate (DCFH-DA) (Beyotime, Haimen, China) according to the previous description [37]. Briefly, 2 × 10^5^ cells per well were seeded into a 12-well plate. After 16 h, the cells were pretreated with quercetin at 7.5, 15, and 30 μM for 2 h, followed by co-treatment with or without QCT at 5 μg/mL. After incubation for 24 h, L02 cells were washed twice with PBS, then the cells were incubated with 500 μL DMEM containing 10 μM DCFH-DA, then the cells were incubated for 30 min at 37 °C in the dark. After three washes with PBS, the cells were observed by fluorescence microscopy (excitation wavelength: 488 nm; emission wavelength: 525 nm). The fluorescence values in each group were measured using Image J software.

The biomarkers of oxidative stress, including the levels of intracellular malondialdehyde (MDA), superoxide dismutase (SOD), catalase (CAT), and reduced glutathione glutathione (GSH) were measured by using commercially available MDA, SOD, CAT, and GSH kits according to the manufacturer’s instructions (Nanjing Jiancheng Biological Engineering, Nanjing, China), respectively. The protein concentration of each sample was determined by BCA assay (Thermo Fisher Scientific Inc, Waltham, MA, USA). The levels of intracellular MDA, SOD, and CAT of each sample were finally normalized to each protein concentration, respectively.

### 2.8. Measurement of Mitochondrial Membrane Potential (MMP)

The changes in mitochondrial membrane potential were examined by using rhodamine-123 staining according to methods described in our previous study [10]. In brief, L02 cells at a density of 2 × 10^5^ cells/well were plated into a 12-well plate and pretreated with quercetin at concentrations of 7.5, 15, and 30 μM for 2 h, followed by treatment with or without QCT at 5 μg/mL for an additional 24 h. After incubation, L02 cells were washed twice with PBS, then incubated with 500 μL DMEM containing 1 μg/mL Rh123 dye at 37 °C in the dark. After three washes with PBS, the cells were digested with 0.25% trypsin-EDTA and collected. A total of 1 × 10^4^ cells were counted in each group and fluorescence values were measured using a microplate fluorescence reader (excitation wavelength: 488 nm; emission wavelength: 525 nm) (Tecan Trading AG, Männedorf, Switzerland).

To further confirm the protective effect of quercetin on QCT-induced loss of mitochondrial membrane potential, similar treatments and measurements were also performed in HepG2 cells.

### 2.9. Measurement of Levels of Cytochrome C and Caspases-3 and -9 Activity

The levels of cytochrome C (CytC), and caspases-3 and -9 activity were measured using the commercially available CytC (Thermo Fisher, Waltham, MA, USA, caspase-9, and caspase-3 (Beyotime, Haimen, China) kits according to commercial instructions. In brief, L02 cells at a density of 2 × 10^5^ cells/well were plated into a 12-well plate and pretreated with quercetin at concentrations of 7.5, 15, and 30 μM for 2 h, followed by treatment with or without QCT at 5 μg/mL for an additional 24 h. Then, L02 cells were washed with ice-cold PBS and lysed using the cell lysis buffer provided by the manufacturer. The collected lysates were centrifuged at 14,000× *g* at 4 °C for 10 min. The supernatants were used to measure the levels of CytC, and the activity of caspases- 9 and -3. Finally, the values of CytC levels and caspases-9 and -3 activity were normalized to the protein concentrations.

### 2.10. Quantitative Reverse-Transcription (qRT)-PCR Examination

After treatment, the cells were washed with PBS and collected for RNA isolation. Total RNAs were isolated using a total RNA isolation kit according to the manufacturer’s instructions (No. RC112-01, Vazyme Biotech Co., Ltd., Nanjing, China). A total of 2 μg RNA was used for the synthesis of cDNA using the Prime Script RT-PCR kit (Takara, Dalian, China). The quality of RNA was assessed by evaluating the optical density (OD) of 260 nm/280 nm. The PCR primers’ information of the Nrf2, HO-1 and β-actin genes followed those used in our previous study [10] and documented in Suppl. Table S2. QRT-PCR was performed using an AB7500 real-time PCR instrument (Applied Biosystems, Foster City, CA, USA). The fold change in gene expression was calculated using the 2^−ΔΔCt^ method and normalized to β-actin.

### 2.11. Western Blotting

L02 cells were pre-treated with quercetin at a final concentration of 30 μM for 2 h, followed by treatment with QCT at 2.5, 5, and 7.5 μg/mL for an additional 24 h. After treatment, the cells were washed with PBS and collected. After treatment, the cells were lysed using a RIPA lysis buffer (Beyotime, Haimen, China) with a protease inhibitor cocktail (1 μg/mL leupeptin, 1 μg/mL pepstatin A, 1 μg/mL aprotinin, and 1 mM PMSF) for 15 min at 4 ˚C, then the cell samples were ultrasonicated using an Ultrasonic Processor (Branson, MO, USA). Finally, the samples were centrifuged at 12,000 rpm at 4 °C for 15 min. The supernatants were collected and the protein concentrations were determined using the BCA assay kit. A total of 20 μg protein from each sample was loaded into a sodium dodecyl sulfate-polyacrylamide gel (SDS-PAGE) and separated electrophoretically, then transferred to a nitrocellulose membrane. The membranes were blocked with non-fat milk for 2 h. After being washed with tris buffered saline tween (TBST), the membranes were incubated with specific primary and secondary antibodies. Rabbit polyclonal antibodies against phosphor (p)-p38 (1:1000 dilution; Cell Signaling Technology, Beverly, MA, USA), HO-1, PARP1, Nrf2, Bax, and Bcl-2 (1:1000 dilution; Proteintech, Chicago, IL, USA), and mouse monoclonal antibody against β-actin (1:1000 dilution; Santa Cruz, CA, USA) were employed. The corresponding anti-mouse or rabbit horseradish peroxidase-conjugated secondary antibodies (1:10,000 dilution, Santa Cruz Biotechnology, Inc., Dallas, TX, USA) were employed. The protein expression in each sample was normalized to β-actin. The values of each blot were analyzed using Image J (National Institute of Mental Health, Bethesda, MD, USA).

Furthermore, the effects of quercetin supplementation on QCT-induced protein expression were confirmed in HepG2 cells. In brief, HepG2 cells were treated with quercetin at final concentrations of 7.5, 15, and 30 μM for 2 h, followed by treatment with QCT at the dose of 10 μg/mL for an additional 24 h. The expression of HO-1, Nrf2, and p-p38 was examined.

### 2.12. Statistical Analysis

All data are shown as mean ± SD. Statistical analysis was analyzed with a one-way analysis of variance (ANOVA), followed by an LSD post hoc test. The *p*-value < 0.05 was considered significant.

## 3. Results

### 3.1. Quercetin Supplementation Attenuates QCT-Induced Cytotoxicity

**Figure 2:**
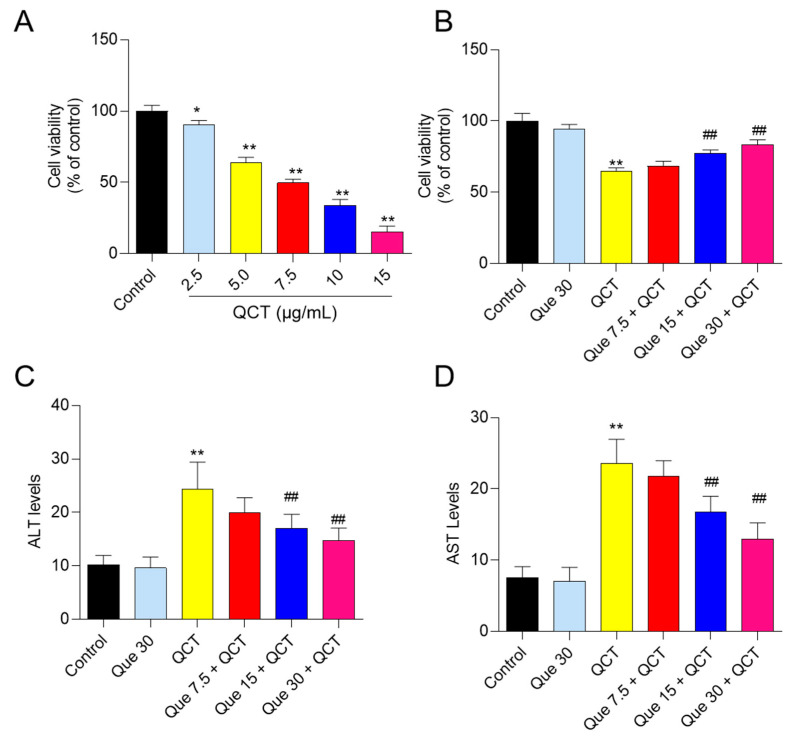
The effect of QCT exposure on cell viabilities and the protective effect of quercetin supplementation in human L02 cells. (**A**), L02 cells were treated with QCT at various concentrations (i.e., 2.5, 5, 7.5, 10, and 15 μg/mL) for 24 h, and cell viabilities were measured using the CCK-8 method. (**B**), L02 cells were pretreated with quercetin at final concentrations of 7.5, 15, and 30 μM for 2 h, followed by co-treatment with QCT at final concentrations of 5 μg/mL for an additional 24 h. Finally, cell viabilities were measured. (**C**,**D**), the effect of quercetin supplementation on the levels of ALT (**C**) and AST (**D**) activity in the culture medium of human L02 cells. All results were presented as mean ± SD, from three independent experiments (*n* = 3). * *p* < 0.05, ** *p* < 0.01, compared to the vehicle control group; ^##^ *p* < 0.01, compared to the QCT-alone group. QCT, quinocetone; Que, quercetin.

In L02 cells, QCT treatment significantly reduced cell viability in a dose-dependent manner when compared to the control group. QCT treatment at concentrations of 2.5, 5, 7.5, 10 and 15 μg/mL for 24 h decreased the viabilities of L02 cells to 90.3%, 64.2%, 49.8%, 34.1% and 15.3% (all *p* < 0.05 or 0.01), respectively (Figure 2A). Quercetin supplementation at 7.5–30 μM significantly improved the QCT-induced decrease in cell viability (Figure 2B); the cell viabilities increased to 68.4%, 77.3% (*p* < 0.05), and 83.2% (*p* < 0.01), respectively, compared to QCT-alone treatment (at a final concentration of 5 μg/mL). Similarly, in HepG2 cells, cell viability assay and morphological observation both showed that quercetin could improve QCT-induced cytotoxicity in a dose-dependent manner (Suppl. Figure S1).

The levels of ALT and AST in the culture medium of L02 cells were measured. As shown in Figure 2C, QCT treatment at 5 μg/mL significantly increased the levels of AST and ALT to 23.6 U/L and 24.4 U/L (both *p* < 0.01), respectively, when compared with that in the control group. Quercetin supplementation significantly inhibited the release of intracellular AST and ALT. Compared with the QCT-alone treatment group, quercetin supplementation at concentrations of 7.5, 15, and 30 μM decreased the levels of ALT to 19.7 U/L, 17.2 U/L (*p* < 0.01), and 14.8 U/L (*p* < 0.01), respectively (Figure 2C) and decreased the levels of AST to 21.8 U/L, 16.8 U/L (*p* < 0.01), and 13.0 U/L (*p* < 0.01), respectively (Figure 2D). Quercetin-alone treatment at 30 μM had no effect on the levels of ALT and AST in the culture medium.

### 3.2. Quercetin Supplementation Attenuates QCT-Induced Apoptosis and DNA Damage

**Figure 3:**
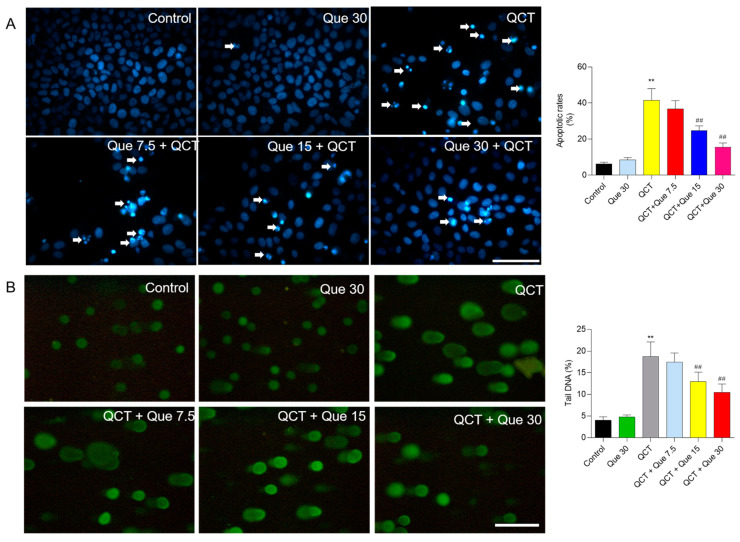
The effect of quercetin supplementation on QCT-induced cell apoptosis and DNA damage in human L02 cells. (**A**), the representative images of cell apoptosis and the results of quantitative analysis. L02 cells were treated with quercetin pretreatment at concentrations of 7.5, 15, and 30 μM for 2 h, followed by co-treatment with QCT at a final concentration of 5 μg/mL for an additional 24 h, and cell apoptosis was stained with Hoechst 33342. Finally, the representative images were obtained using the fluorescence microscope (on the left) and apoptotic rates were quantified using Image J (on the right). The white arrowheads indicate the apoptotic cells. (**B**), the results of the comet assay. L02 cells were pretreated with quercetin at final concentrations of 7.5, 15, and 30 μM for 2 h, followed by co-treatment with QCT at a final concentration of 5 μg/mL for 4 h, and the comet assay was performed. The representative images were obtained using the fluorescence microscope (on the left) and the percentage (%) of tail DNA was quantified (on the right). All results were presented as mean ± SD (*n* = 4 independent experiments). ** *p* < 0.01, compared to the vehicle control group; ^##^ *p* < 0.01, compared to the QCT-alone group. QCT, quinocetone; Que, quercetin; Bar = 50 μm.

As shown in Figure 3A, compared with the control group, QCT treatment at 5 μg/mL for 24 h induced significant chromosomal aggregation and nuclear fragmentation in L02 cells, and the cell apoptosis rates increased to 41.5% (*p* < 0.01). Quercetin administration at 7.5, 15, and 30 μM substantially prevented QCT-induced cell death, with cell apoptosis rates decreasing to 36.7%, 24.8% (*p* < 0.01), and 15.5% (*p* < 0.01), respectively, when compared with the QCT-alone treatment group. Consistently, quercetin supplementation at 7.5–30 μM treatment also significantly improved QCT-induced apoptosis in HepG2 cells (Suppl. Figure S2).

Furthermore, the comet assay was performed to assess the protective effect of quercetin on the DNA damage caused by QCT treatment in L02 cells. As shown in Figure 3B, compared with the control, QCT treatment at 5 μg/mL for 4 h significantly increased the percentage (%) of tail DNA to 18.8% (*p* < 0.01). Quercetin treatment at 15 and 30 μM significantly decreased the % of tail DNA to 13.1% and 10.5% (both *p* < 0.01), respectively, when compared with the QCT-alone treatment group. Quercetin-alone treatment at 30 μM did not result in cell apoptosis and DNA damage in L02 cells and HepG2 cells (Figure 3 and Suppl. Figure S2).

### 3.3. Quercetin Attenuates QCT-Induced Production of ROS and Oxidative Damage

**Figure 4:**
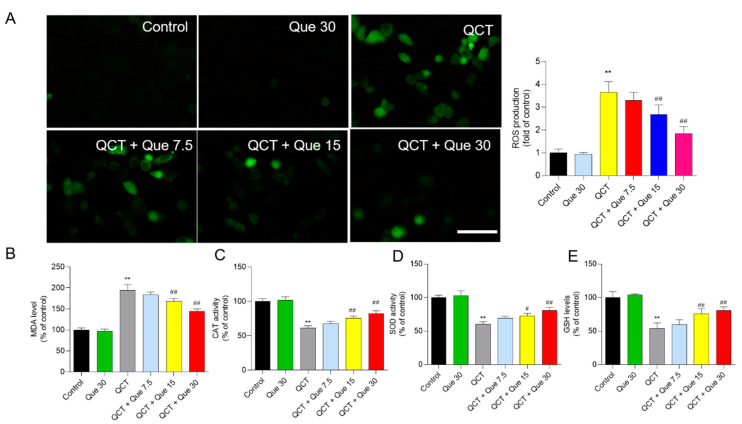
The effect of quercetin supplementation on QCT-induced ROS production and oxidative damage in human L02 cells. (**A**), Measurement of ROS production. Intracellular ROS production was measured using the dye 2,7-dichlorofluorescein diacetate staining. The representative images were shown (on the left) and the fluorescence intensities were quantified using Image J (on the right). Bar = 50 μm. (**B**–**E**), The effect of quercetin supplementation on MDA levels (**B**), catalase (CAT) activity (**C**), superoxide dismutase (SOD) activity (**D**), and glutathione (GSH) levels (**E**) were measured, respectively. All results were presented as mean ± SD (*n* = 4 independent experiments). ** *p* < 0.01, compared to the vehicle control group; ^#^ *p* < 0.05, and ^##^ *p* < 0.01, compared to the QCT-alone group. QCT, quinocetone; Que, quercetin; Bar = 50 μm.

QCT treatment significantly elevated the production of intracellular ROS, which was partly inhibited by quercetin co-treatment in a dose-dependent manner. As shown in Figure 4A, in L02 cells, QCT treatment at 5 μg/mL significantly increased the intracellular ROS levels by 3.7-fold, when compared with the control group. Quercetin treatment at concentrations of 7.5, 15, and 30 μM significantly decreased the intracellular ROS levels by 3.3-, 2.7- (*p* < 0.01), and 1.9-fold (*p* < 0.01), when compared with the QCT-alone treatment group. Similarly, quercetin supplementation at concentrations of 7.5, 15, and 30 μM also significantly inhibited the QCT-induced production of ROS in HepG2 cells (Suppl. Figure S3).

The biomarkers of oxidative stress were further assessed in L02 cells. Our results showed that quercetin supplementation significantly improved QCT-induced oxidative damage. As shown in Figure 4B–D, when compared with the QCT-alone treatment group, quercetin treatment at concentrations of 7.5, 15, and 30 μM significantly decreased the levels of MDA from 194.5% to 183.8%, 168.2% (*p* < 0.01), and 139.9% (*p* < 0.01), respectively; increased the CAT activities from 61.3% to 67.8%, 75.8% (*p* < 0.01), and 82.3%(*p* < 0.01), respectively; increased the SOD activities from 60.2% to 69.3%, 72.5% (*p* < 0.05), and 80.6% (*p* < 0.01), respectively; and increased the GSH levels from 54.0% to 59.8%, 75.8% (*p* < 0.05), and 80.5% (*p* < 0.01), respectively.

### 3.4. Quercetin Attenuates QCT-Induced Mitochondrial Dysfunction

**Figure 5:**
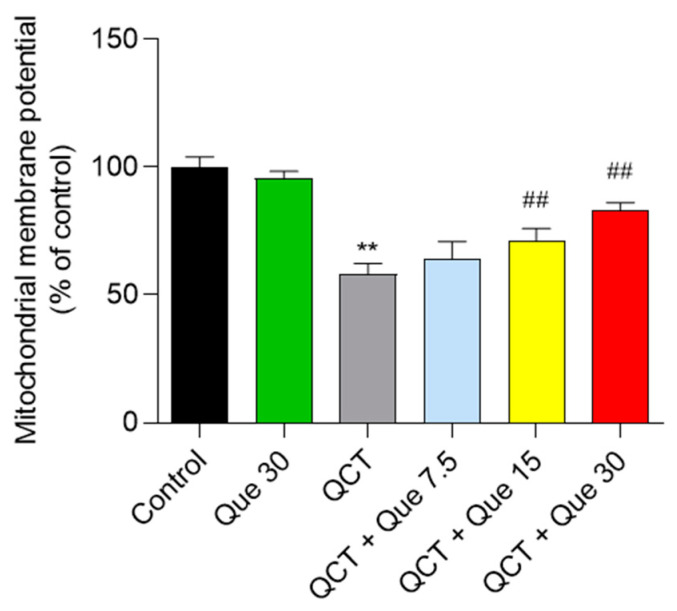
The effects of quercetin supplementation on QCT-induced loss of mitochondrial membrane potential (MMP) in L02 cells. All results were presented as mean ± SD (*n* = 4 independent experiments). ** *p* < 0.01, compared to the vehicle control group; ^##^ *p* < 0.01, compared to the QCT-alone group. QCT, quinocetone; Que, quercetin.

In L02 cells, QCT treatment at 5 μg/mL significantly induced the loss of MMP, which was significantly alleviated by the quercetin supplement. As shown in Figure 5, QCT treatment decreased MMP to 58.1% (*p* < 0.01), when compared with the control group. Quercetin treatment at concentrations of 7.5, 15, and 30 μM significantly increased MMP to 64.2%, 72.3% (*p* < 0.01), and 82.8% (*p* < 0.01), respectively, when compared with the QCT-alone treatment group. Consistently, quercetin treatment at concentrations of 7.5, 15, and 30 μM also significantly improved the QCT-induced loss of MMP in HepG2 cells (Suppl. Figure S4). In human L02 cells and HepG2 cells, quercetin administration at 30 μM had no effect on MMP when compared with the control group (Figure 5 and Suppl. Figure S4).

### 3.5. Quercetin Attenuates the QCT-Induced Activation of the Mitochondrial Apoptotic Pathway

**Figure 6:**
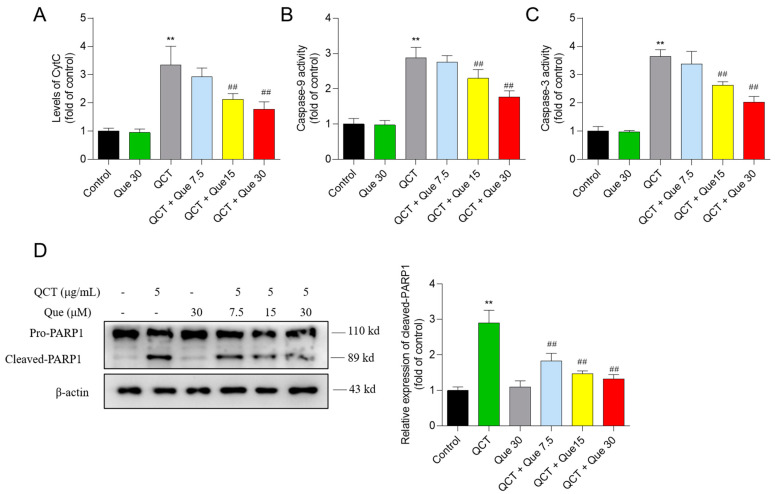
The changes of CytC levels (**A**), caspase-9 (**B**) and caspase -3 (**C**) activity, and cleaved-PARP1 protein expression (**D**). All results were presented as mean ± SD (*n* = 4 independent experiments). ** *p* < 0.01, compared to the vehicle control group; ^##^ *p* < 0.01, compared to the QCT-alone group. QCT, quinocetone; Que, quercetin.

QCT treatment at 5 μg/mL substantially upregulated CytC protein expression, caspases-9 activity, caspase-3 activity, and cleaved PARP1 protein expression to 3.4-, 2.9-, 3.7-, and 2.9-fold (all *p* < 0.01), respectively, when compared with the control (Figure 6). Moreover, compared to the QCT-alone treatment group, quercetin co-treatment at 15 and 30 μM significantly decreased the CytC levels to 2.1- and 1.8-fold (both *p* < 0.01), respectively (Figure 6A); decreased the caspase-9 activity to 2.3- and 1.8-fold (both *p* < 0.01), respectively (Figure 6B); decreased the caspase-3 activity to 2.7- and 2.0-fold (both *p* < 0.01), respectively (Figure 6C); and decreased the cleaved-PARP1 protein expression to 1.5- and 1.3-fold (both *p* < 0.01), respectively (Figure 6D). There were no significant changes in the levels of CytC, the activity of caspases-9 and -3, and the cleaved-PARP1 protein expression in the quercetin-alone treatment, when compared with the control group (Figure 6).

### 3.6. Effects of Quercetin Supplementation on the Expression of Nrf2, HO-1, p-p38, Bcl-2, and Bax Proteins

**Figure 7:**
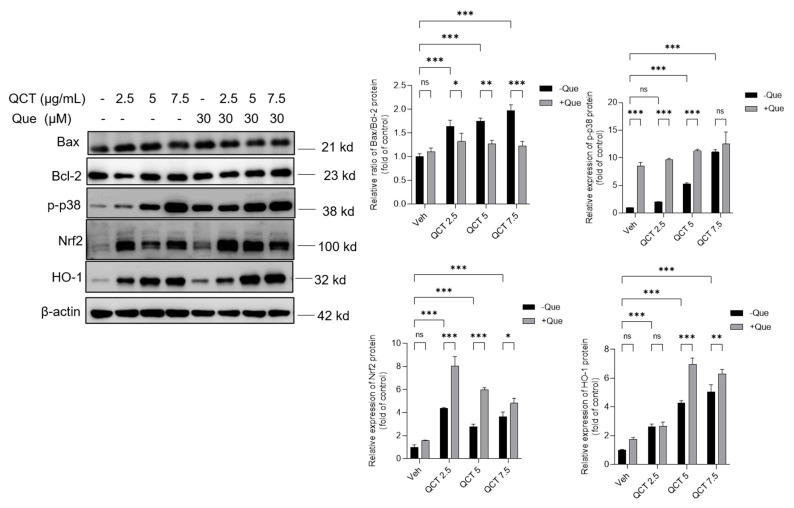
Quercetin supplementation upregulated the expression of Nrf2, HO-1, p-p38, and Bcl-2 proteins and downregulated Bax proteins in human L02 cells. QCT-alone treatment was performed at final concentrations of 2.5, 5, and 7.5 μg/mL with or without quercetin at a final concentration of 30 μM for 24 h, and the expression of Bax, Bcl-2, p-p38, Nrf2, and HO-1 proteins was examined using the Western blot method. The representative images were shown (on the left) and the values of each band were quantified using Image J (on the right). All results were presented as mean ± SD (*n* = 3 independent experiments). * *p* < 0.05, ** *p* < 0.01, and *** *p* < 0.001, compared between the two groups. QCT, quinocetone; Que, quercetin; ns, no significance.

As shown in Figure 7, in L02 cells, QCT treatment at 2.5–7.5 μg/mL increased the expression of Bax, p-p38, Nrf2, and HO-1 proteins while downregulated the expression of Bcl-2 protein in a dose-dependent manner. When compared with the control group, QCT treatment at 2.5, 5, and 7.5 μg/mL increased the ratio of Bax/Bcl-2 to 1.6-, 1.7- (*p* < 0.01), and 2.0-fold (*p* < 0.01), respectively; increased the expression of p-p38 protein to 2.1-, 5.3-(*p* < 0.01), and 11.3- fold (*p* < 0.01), respectively; increased the expression of Nrf2 protein to 4.3-, 2.7-, and 3.6- fold (all *p* < 0.01), respectively; and increased the expression of HO-1 protein to 2.6-, 4.2-, and 5.1- fold (all *p* < 0.01), respectively (Figure 7). Quercetin treatment at 30 μM significantly upregulated the expression of Nrf2 and HO-1 proteins, and significantly downregulated the ratios of Bax/Bcl-2, when compared with each QCT-alone treatment group. When compared with the vehicle control group, quercetin-alone treatment at 30 μM increased the expression of p-p38, Nrf2, and HO-1 proteins by 8.5-, 1.6-, and 1.8-fold, respectively (Figure 7).

Consistently, compared to the QCT-alone treatment group, quercetin treatment at concentrations of 7.5, 15, and 30 μM significantly increased QCT-induced expressions of these mentioned proteins in a dose-dependent manner in HepG2 cells (Suppl. Figure S5). Quercetin-alone treatment at 30 μM significantly increased the expressions of Nrf2, HO-1 and p-p38 proteins (all *p* < 0.01), when compared with the untreated cells (Suppl. Figure S5).

### 3.7. Pharmacological Inhibition of the p38 or Nrf2 Pathways Partly Attenuates the Protective Effect of Quercetin Supplementation on QCT-Induced Cytotoxicity

**Figure 8:**
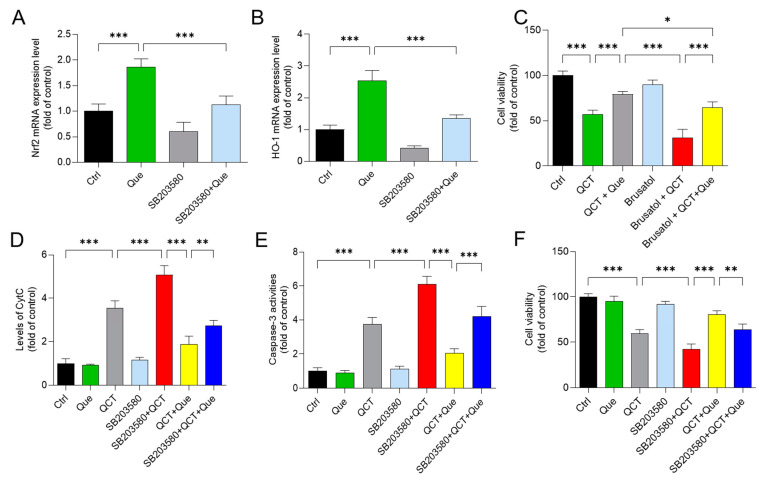
Inhibition of the p38 pathway attenuates the protective effect of quercetin on QCT-induced cytotoxicity in human L02 cells. A and B, the effect of p38 inhibitor treatment (i.e., SB203580 at 10 μM) on the quercetin (at 30 μM) -induced expression of Nrf2 (**A**) and HO-1 (**B**) mRNAs. (**C**), L02 cells were pretreated with Que at 30 μM or co-treated brusatol at 40 nM for 2 h, followed by treatment with or without QCT at a final concentration of 5 μg/mL for an additional 24 h. Finally, the cell viability was measured. D-F, L02 cells were pretreated with Que at 30 μM or co-treated SB203580 at 10 μM for 2 h, followed by treatment with or without QCT at a final concentration of 5 μg/mL for an additional 24 h. Finally, the release of CytC (**D**), the activation of caspase-3 (**E**), and cell viabilities (**F**) were measured, respectively. All results were presented as mean ± SD (*n* = 4 independent experiments). * *p* < 0.05, ** *p* < 0.01, and *** *p* < 0.001, compared between the two groups. QCT, quinocetone; Que, quercetin.

As shown in Figure 8, in L02 cells, inhibition of p38 by SB203580 significantly attenuated the mRNA expression of Nrf2 and HO-1 induced by quercetin treatment (Figure 8A,B). Moreover, pharmacological inhibition of Nrf2 by brusatol (at 40 nM) significantly promoted QCT-induced cytotoxicity and reduced the protective effects of quercetin (Figure 8C). Furthermore, pharmacological inhibition of p38 significantly increased the QCT-induced release of CytC, and caspase-3 activity, finally exacerbated QCT-induced cytotoxicity and reduced the protective effect of quercetin supplementation (Figure 8D–F).

## 4. Discussion

Many members of the QdNOs family have been employed in medicinal and agricultural industries due to their powerful biological activity [38]. Since 2003, QCT, one of the quinoxaline 1,4-di-N-Oxide derivatives, has been approved in China to treat bacterial infections or as a food additive in veterinary medicine [38,39]. However, recent research indicated that QCT could induce DNA damage and genotoxicity *in vitro* and in animal models [9,11,40,41]. QCT or metabolites might be accumulated in the human body through food consumption, posing a potential risk to human health [42]. Therefore, a deep understanding of the precise molecular mechanisms or effective toxicity prevention strategies of QCT are required for the risk assessment and combination usage.

The current work investigated the cytotoxicity of QCT and its underlying molecular mechanisms in human L02 cells and HepG2 cells. Our findings indicated that QCT exposure at the low dose range (i.e., at 2.5–15 μg/mL) could result in marked oxidative stress damage, mitochondrial dysfunction, caspase activation, and cell apoptosis in human L02 cells and HepG2 cells (Figure 2, Figure 3, Figure 4 and Figure 5 and Suppl. Figures S1–S4). These findings are consistent with the previous report on QCT’s cytotoxicity [10,17,18,19,20,22,23,24]. Furthermore, for the first time, our current study found that quercetin, a flavonoid compound derived from a variety of vegetables and Chinese herbs, could significantly improve QCT exposure-induced cytotoxicity, oxidative stress, and apoptosis. Mechanistically, quercetin may enhance QCT-induced cytotoxicity and apoptosis by activating the p38/Nrf2/HO-1 pathway and inhibiting ROS generation (Figure 2, Figure 3, Figure 4, Figure 5, Figure 6, Figure 7 and Figure 8 and Suppl. Figures S1–S5).

Multiple studies have shown that excessive ROS accumulation may play a critical role in QCT-induced cytotoxicity and tissue damage [9,12,16,17,18,20,34]. It has been demonstrated that QCT-induced intracellular ROS accumulation is associated with its special structure, i.e., the N-oxide group of QCT, and its reduction by the CYP450 enzyme in the liver could cause the production of ROS [5,9,43]. This is because bidesoxy-quinocetone, the major metabolite of QCT, exhibited fewer toxic effects than QCT per se in vitro [5]. The endogenous antioxidant enzymes or antioxidants could neutralize ROS and play critical roles in maintaining redox homeostasis [10,16]. Some studies found that QCT exposure could downregulate the activities of intracellular SOD, CAT, GPx, and the levels of GSH, then aggravate QCT-induced oxidative stress [9,12,16,17,18,20,34]. A previous study reported that Pu-erh black tea supplementation could effectively improve the QCT-induced elevation of AST and ALT, two biomarkers of liver function, and improved the QCT-induced production of ROS and oxidative stress in the liver tissues of mice, indicating that antioxidant supplementation may be a potential strategy to improve QCT-induced adverse effects [24]. Similarly, in the present study, our data showed that quercetin supplementation could significantly inhibit QCT-induced increases in ALT and AST levels and the production of ROS, then improve QCT-induced cytotoxicity in L02 and HepG2 cells (Figure 2 and Figure 4 and Suppl. Figures S1 and S3). Previous studies have shown that quercetin could direct scavenge the common small free radicals, such as HOO^•^, ^•^NO and O_2_^•−^, then inhibit intracellular ROS accumulation [44,45]. In addition, many studies have reported that quercetin supplementation could effectively improve drugs, environmental toxins, and chronic diseases-induced cell or tissue damage by inhibiting the production of ROS, oxidative stress, and cell apoptosis [45,46,47,48,49,50]. Taken together, our results suggest that inhibiting ROS generation and increasing the activity of cellular antioxidant enzymes may contribute to quercetin’s protective impact against QCT-induced cytotoxicity in human L02 cells.

Excessive ROS production in cells could cause damage to DNA, lipid, proteins, and other biomolecules, and finally trigger cell death [34]. Indeed, in the present study, increased apoptosis and DNA damage rates were detected in human L02 and HepG2 cells treated with QCT (Figure 3 and Suppl. Figure S2), which is consistent with our previous studies [10,17,19,21,22,34]. Our previous study showed that inhibition of ROS production could effectively improve QCT-induced DNA damage and apoptosis [10,17,34]. In line with these previous studies, our current data showed that quercetin supplementation could significantly inhibit QCT-induced DNA damage and apoptosis (Figure 3 and Suppl. Figure S2). It indicated that inhibition of ROS production by quercetin could effectively inhibit QCT-induced cell apoptosis.

Mitochondria are not only the main site of ROS production, but are also the target of ROS [51]. Wang et al. found that QCT could target mitochondria and induce mitochondrial DNA, which might affect oxidative phosphorylation and promote QCT-induced ROS production [52,53]. Mitochondria are the most vulnerable targets of ROS and mitochondrial dysfunction may trigger the mitochondrial pathway to induce cell apoptosis [54]. The loss of MMP is an important characteristic indicative of mitochondrial dysfunction [55]. As the data shows, QCT treatment caused a significant loss of MMP in L02 and HepG2 cells (Figure 5 and Suppl. Figure S4). Disrupted membrane potential may promote the opening of mitochondrial permeability transition pores (MPTPs), and induce the release of CytC, and activate the activity of caspase-9 and -3, finally resulting in an increase in cleaved PARP1 and triggering cell apoptosis [54,55]. Bax and Bcl-2 are also two important markers of mitochondrial apoptotic pathways and the increased Bax/Bcl-2 could promote the opening of MPTPs and exacerbate mitochondrial apoptotic pathways [56]. Caspase-9 is also a biomarker of the mitochondrial apoptotic pathway [57]. In the present study, quercetin treatment significantly ameliorated the QCT-induced loss of MMP in L02 and HepG2 cells (Figure 5 and Suppl. Figure S4). Furthermore, it was found that quercetin supplementation could decrease the ratio of Bax/Bcl-2, the release of CytC, and the activation of caspases-9 and -3, and an increase in cleaved-PARP1 caused by QCT exposure in L02 cells (Figure 6 and Figure 7). Several studies have shown that quercetin supplementation could improve diabetes-induced testicular anomaly [58], and d-galactosamine-induced human L02 cell damage [59], and copper-induced apoptotic cell death [60] by inhibiting the oxidative-stress-driven mitochondrial apoptotic pathway. Taken together, this evidence indicated that quercetin could offer a protective effect against QCT-induced apoptosis by inhibiting mitochondrial pathways, which may be related to the inhibitory effect on the production of ROS. The precise molecular mechanism involved in the integration and interaction between oxidative stress and mitochondria needs further investigation.

Nrf2 is an oxidative-stress-mediated transcription factor with a variety of downstream targets aimed at cryoprotection against oxidative stress [61]. To date, it has been demonstrated that Nrf2 could drive the transcription of >300 antioxidant-response-element (ARE)-regulated genes that are involved in many critical cellular processes, including xenobiotic detoxification, redox regulation, proteostasis, and primary metabolism [61]. Indeed, many studies reported that the activation of Nrf2 transcription activity could improve oxidative stress damage caused by drugs or toxins, such as cadmium, colistin, and furazolidone [10,37,54,57,62]. Nevertheless, the activation of Nrf2 could also ameliorate QCT-induced cytotoxicity and tissue damage in vivo and in vitro, which had been confirmed in several studies [10,16,18]. Moreover, a previous study has demonstrated that the inhibition of HO-1 activity could promote QCT-induced cytotoxicity and apoptosis in L02 cells [10]. HO-1 is one of the Nrf2 downstream genes and is an important mediator in the process of Nrf2-transcriptional-activation-mediated anti-oxidative stress [61]. Consistently, in the present study, QCT exposure also significantly increased the expression of Nrf2 and HO-1 proteins in both L02 and HepG2 cells (Figure 7 and Suppl. Figure S5). Quercetin supplementation could further promote the expression of Nrf2 and HO-1 proteins, then inhibit QCT-induced mitochondrial apoptotic cell death (Figure 7 and Suppl. Figure S5). Pharmacological inhibition of Nrf2 could partly block the protective effect of quercetin (Figure 8). This evidence indicates that Nrf2 activation plays a critical role in the protective effect of quercetin against QCT-induced cytotoxicity. However, there is no evidence showing the direct interaction between quercetin and the Nrf2 protein. Several studies showed that the activation of Nrf2 by quercetin may involve p38 signaling [63,64,65]. In line with these previous studies, our data showed quercetin treatment significantly increased the expression of the p-p38 protein in both L02 cells and HepG2 cells (Figure 7 and Suppl. Figure S5). Inhibition of p38 signaling significantly decreased the expression of Nrf2 and HO-1 mRNA in L02 cells (Figure 8). Furthermore, we found that the inhibition of p38 signaling significantly promoted the QCT-induced expression of CytC and caspase-3 in L02 cells, resulting in the attenuation of the protective effect of quercetin (Figure 8). Taken together, our results indicated that the inhibition of the mitochondrial apoptotic pathway by quercetin is partly dependent on the activation of the p38/Nrf2/HO-1 pathway.

A clinical trial also showed that oral quercetin supplementation at 2 g/day within 24 h could reduce the occurring oxidative stress as well as inflammation in sarcoidosis patients [66]. An early study showed that oral quercetin supplementation at 1 g/day for 3 weeks could protect against an exercise-induced inflammatory response and had no unwanted adverse effects in humans [67]. Lu et al. found that oral supplementation at 5 g/day for 28 days showed a protective effect against liver damage caused by the hepatitis C virus in humans and there were no toxic effects on the liver [68]. These data indicate that quercetin supplementation was safe to use in clinical trials.

In conclusion, our study demonstrated that QCT could result in marked oxidative stress, DNA damage, and apoptotic cell death in vitro, which may cause potential health risks to animals or humans. Quercetin supplementation might reduce QCT-induced cell apoptosis in vitro via activating the p38/Nrf2/HO-1 pathway and inhibiting the ROS/mitochondrial apoptotic pathway. Our findings suggest that quercetin may be a promising candidate for preventing and treating QdNOs-mediated harmful effects in humans or animals.

## Data Availability

All of the data is contained within the article and the supplementary materials.

## Supplementary Materials

The following supporting information can be downloaded at: https://www.mdpi.com/article/10.3390/antiox11081498/s1, Suppl. Table S1. STR and amelogenin genotyping results of L02 cell line. Suppl. Table S2. Primer sequences of the quantitative real-time PCR. Suppl. Figure S1. The effect of quercetin supplementation on QCT-induced cytotoxicity in HepG2 cells. Suppl. Figure S2. The effect of quercetin supplementation on QCT-induced cell apoptosis in HepG2 cells. Suppl. Figure S3. Effect of quercetin supplementation on QCT-induced ROS production and oxidative damage in human HepG2 cells. Suppl. Figure S4. Effects of quercetin supplementation on QCT-induced loss of mitochondrial membrane potential (MMP) in HepG2 cells. Suppl. Figure S5. Quercetin supplementation upregulated the expression of Nrf2, HO-1, and p-p38 proteins in human HepG2 cells.

## References

[1] Silva L., Coelho P., Teixeira D., Monteiro A., Pinto G., Soares R., Prudêncio C., Vieira M. (2020). Oxidative Stress Modulation and Radiosensitizing Effect of Quinoxaline-1,4-Dioxides Derivatives. Anti-Cancer Agents Med. Chem..

[2] Le T., Zhu L., Shu L., Zhang L. (2016). Simultaneous determination of five quinoxaline-1,4-dioxides in animal feeds using an immunochromatographic strip. Food Addit. Contam. Part A Chem. Anal. Control Expo. Risk Assess..

[3] Diab-Assef M., Haddadin M.J., Yared P., Assaad C., Gali-Muhtasib H.U. (2002). Quinoxaline 1,4-dioxides: Hypoxia-selective therapeutic agents. Mol. Carcinog..

[4] Buravchenko G.I., Maslov D.A., Alam M.S., Grammatikova N.E., Frolova S.G., Vatlin A.A., Tian X., Ivanov I.V., Bekker O.B., Kryakvin M.A. (2022). Synthesis and Characterization of Novel 2-Acyl-3-trifluoromethylquinoxaline 1,4-Dioxides as Potential Antimicrobial Agents. Pharmaceuticals.

[5] Wang X., Wan D., Ihsan A., Liu Q., Cheng G., Li J., Liu Z., Yuan Z. (2015). Mechanism of adrenocortical toxicity induced by quinocetone and its bidesoxy-quinocetone metabolite in porcine adrenocortical cells in vitro. Food Chem. Toxicol..

[6] Li J., Huang L., Wang X., Pan Y., Liu Z., Chen D., Tao Y., Wu Q., Yuan Z. (2014). Metabolic disposition and excretion of quinocetone in rats, pigs, broilers, and carp. Food Chem. Toxicol..

[7] Wang X., Zhang W., Wang Y.L., Ihsan A., Dai M.H., Huang L.L., Chen D.M., Tao Y.F., Peng D.P., Liu Z.L. (2012). Two generation reproduction and teratogenicity studies of feeding quinocetone fed to Wistar rats. Food Chem. Toxicol..

[8] Wang X., Zhang W., Wang Y., Peng D., Ihsan A., Huang X., Huang L., Liu Z., Dai M., Zhou W. (2010). Acute and sub-chronic oral toxicological evaluations of quinocetone in Wistar rats. Regul. Toxicol. Pharmacol. RTP.

[9] Zhang K., Zheng W., Zheng H., Wang C., Wang M., Li T., Wang X., Zhang L., Xiao S., Fei C. (2014). Identification of oxidative stress and responsive genes of HepG2 cells exposed to quinocetone, and compared with its metabolites. Cell Biol. Toxicol..

[10] Dai C., Li B., Zhou Y., Li D., Zhang S., Li H., Xiao X., Tang S. (2016). Curcumin attenuates quinocetone induced apoptosis and inflammation via the opposite modulation of Nrf2/HO-1 and NF-kB pathway in human hepatocyte L02 cells. Food Chem. Toxicol..

[11] Chen Q., Tang S., Jin X., Zou J., Chen K., Zhang T., Xiao X. (2009). Investigation of the genotoxicity of quinocetone, carbadox and olaquindox in vitro using Vero cells. Food Chem. Toxicol..

[12] Yang W., Fu J., Xiao X., Yan H., Bao W., Wang D., Hao L., Nussler A.K., Yao P., Liu L. (2013). Quinocetone triggers oxidative stress and induces cytotoxicity and genotoxicity in human peripheral lymphocytes of both genders. J. Sci. Food Agric..

[13] Wang X., Bai Y., Cheng G., Ihsan A., Zhu F., Wang Y., Tao Y., Chen D., Dai M., Liu Z. (2016). Genomic and proteomic analysis of the inhibition of synthesis and secretion of aldosterone hormone induced by quinocetone in NCI-H295R cells. Toxicology.

[14] Ihsan A., Wang X., Zhang W., Tu H., Wang Y., Huang L., Iqbal Z., Cheng G., Pan Y., Liu Z. (2013). Genotoxicity of quinocetone, cyadox and olaquindox in vitro and in vivo. Food Chem. Toxicol. Int. J. Publ. Br. Ind. Biol. Res. Assoc..

[15] Jin X., Chen Q., Tang S.S., Zou J.J., Chen K.P., Zhang T., Xiao X.L. (2009). Investigation of quinocetone-induced genotoxicity in HepG2 cells using the comet assay, cytokinesis-block micronucleus test and RAPD analysis. Toxicol. Vitr. Int. J. Publ. Assoc. BIBRA.

[16] Yu M., Wang D., Xu M., Liu Y., Wang X., Liu J., Yang X., Yao P., Yan H., Liu L. (2014). Quinocetone-induced Nrf2/HO-1 pathway suppression aggravates hepatocyte damage of Sprague-Dawley rats. Food Chem. Toxicol..

[17] Yang X., Tang S., Dai C., Li D., Zhang S., Deng S., Zhou Y., Xiao X. (2017). Quinocetone induces mitochondrial apoptosis in HepG2 cells through ROS-dependent promotion of VDAC1 oligomerization and suppression of Wnt1/β-catenin signaling pathway. Food Chem. Toxicol..

[18] Yu M., Xu M., Liu Y., Yang W., Rong Y., Yao P., Yan H., Wang D., Liu L. (2013). Nrf2/ARE is the potential pathway to protect Sprague-Dawley rats against oxidative stress induced by quinocetone. Regul. Toxicol. Pharmacol. RTP.

[19] Zhou Y., Zhang S., Dai C., Tang S., Yang X., Li D., Zhao K., Xiao X. (2016). Quinocetone triggered ER stress-induced autophagy via ATF6/DAPK1-modulated mAtg9a trafficking. Cell Biol. Toxicol..

[20] Zhang C., Wang C., Tang S., Sun Y., Zhao D., Zhang S., Deng S., Zhou Y., Xiao X. (2013). TNFR1/TNF-α and mitochondria interrelated signaling pathway mediates quinocetone-induced apoptosis in HepG2 cells. Food Chem. Toxicol..

[21] Zhang S., Zhang C., Tang S., Deng S., Zhou Y., Dai C., Yang X., Xiao X. (2016). AKT/TSC2/p70S6K signaling pathway is involved in quinocetone-induced death-promoting autophagy in HepG2 cells. Toxicol. Mech. Methods.

[22] Zhou Y., Zhang S., Deng S., Dai C., Tang S., Yang X., Li D., Zhao K., Xiao X. (2016). ML-7 amplifies the quinocetone-induced cell death through akt and MAPK-mediated apoptosis on HepG2 cell line. Toxicol. Mech. Methods.

[23] Wang D., Luo X., Zhong Y., Yang W., Xu M., Liu Y., Meng J., Yao P., Yan H., Liu L. (2012). Pu-erh black tea extract supplementation attenuates the oxidative DNA damage and oxidative stress in Sprague-Dawley rats with renal dysfunction induced by subchronic 3-methyl-2-quinoxalin benzenevinylketo-1,4-dioxide exposure. Food Chem. Toxicol..

[24] Wang D., Zhong Y., Luo X., Wu S., Xiao R., Bao W., Yang W., Yan H., Yao P., Liu L. (2011). Pu-erh black tea supplementation decreases quinocetone-induced ROS generation and oxidative DNA damage in Balb/c mice. Food Chem. Toxicol..

[25] Andres S., Pevny S., Ziegenhagen R., Bakhiya N., Schäfer B., Hirsch-Ernst K.I., Lampen A. (2018). Safety Aspects of the Use of Quercetin as a Dietary Supplement. Mol. Nutr. Food Res..

[26] Reyes-Farias M., Carrasco-Pozo C. (2019). The Anti-Cancer Effect of Quercetin: Molecular Implications in Cancer Metabolism. Int. J. Mol. Sci..

[27] Marunaka Y., Marunaka R., Sun H., Yamamoto T., Kanamura N., Inui T., Taruno A. (2017). Actions of Quercetin, a Polyphenol, on Blood Pressure. Molecules.

[28] Colunga Biancatelli R.M.L., Berrill M., Catravas J.D., Marik P.E. (2020). Quercetin and Vitamin C: An Experimental, Synergistic Therapy for the Prevention and Treatment of SARS-CoV-2 Related Disease (COVID-19). Front. Immunol..

[29] Shi T., Bian X., Yao Z., Wang Y., Gao W., Guo C. (2020). Quercetin improves gut dysbiosis in antibiotic-treated mice. Food Funct..

[30] Sanjay S., Girish C., Toi P.C., Bobby Z. (2021). Quercetin modulates NRF2 and NF-κB/TLR-4 pathways to protect against isoniazid- and rifampicin-induced hepatotoxicity in vivo. Can. J. Physiol. Pharmacol..

[31] Gao S., Duan X., Wang X., Dong D., Liu D., Li X., Sun G., Li B. (2013). Curcumin attenuates arsenic-induced hepatic injuries and oxidative stress in experimental mice through activation of Nrf2 pathway, promotion of arsenic methylation and urinary excretion. Food Chem. Toxicol. Int. J. Publ. Br. Ind. Biol. Res. Assoc..

[32] Cao J., Liu Y., Jia L., Jiang L.P., Geng C.Y., Yao X.F., Kong Y., Jiang B.N., Zhong L.F. (2008). Curcumin attenuates acrylamide-induced cytotoxicity and genotoxicity in HepG2 cells by ROS scavenging. J. Agric. Food Chem..

[33] Kim M., Jee S.C., Kim K.S., Kim H.S., Yu K.N., Sung J.S. (2021). Quercetin and Isorhamnetin Attenuate Benzo[a]pyrene-Induced Toxicity by Modulating Detoxification Enzymes through the AhR and NRF2 Signaling Pathways. Antioxidants.

[34] Dai C., Tang S., Li D., Zhao K., Xiao X. (2015). Curcumin attenuates quinocetone-induced oxidative stress and genotoxicity in human hepatocyte L02 cells. Toxicol. Mech. Methods.

[35] Dai C., Li H., Wang Y., Tang S., Velkov T., Shen J. (2021). Inhibition of Oxidative Stress and ALOX12 and NF-κB Pathways Contribute to the Protective Effect of Baicalein on Carbon Tetrachloride-Induced Acute Liver Injury. Antioxidants.

[36] Li D., Dai C., Yang X., Li B., Xiao X., Tang S. (2017). GADD45a Regulates Olaquindox-Induced DNA Damage and S-Phase Arrest in Human Hepatoma G2 Cells via JNK/p38 Pathways. Molecules.

[37] Dai C., Ciccotosto G.D., Cappai R., Tang S., Li D., Xie S., Xiao X., Velkov T. (2018). Curcumin Attenuates Colistin-Induced Neurotoxicity in N2a Cells via Anti-inflammatory Activity, Suppression of Oxidative Stress, and Apoptosis. Mol. Neurobiol..

[38] Rivera G. (2022). Quinoxaline 1,4-di-N-Oxide Derivatives: Are They Unselective or Selective Inhibitors?. Mini Rev. Med. Chem..

[39] Zhao Y., Cheng G., Hao H., Pan Y., Liu Z., Dai M., Yuan Z. (2016). In vitro antimicrobial activities of animal-used quinoxaline 1,4-di-N-oxides against mycobacteria, mycoplasma and fungi. BMC Vet. Res..

[40] Hou L., Liu F., Zhao C., Fan L., Hu H., Yin S. (2022). Combination of oxytetracycline and quinocetone synergistically induces hepatotoxicity via generation of reactive oxygen species and activation of mitochondrial pathway. Toxicol. Mech. Methods.

[41] Wang X., Zhang H., Huang L., Pan Y., Li J., Chen D., Cheng G., Hao H., Tao Y., Liu Z. (2015). Deoxidation rates play critical role in DNA damage of important synthetic drugs, quinoxaline 1,4-dioxides. Chem. Res. Toxicol..

[42] Li J., Huang L., Pan Y., Chen D., Wang X., Ahmad I., Tao Y., Liu Z., Yuan Z. (2014). Tissue depletion of quinocetone and its five major metabolites in pigs, broilers, and carp fed quinocetone premix. J. Agric. Food Chem..

[43] Wang X., Martínez M.A., Cheng G., Liu Z., Huang L., Dai M., Chen D., Martínez-Larrañaga M.R., Anadón A., Yuan Z. (2016). The critical role of oxidative stress in the toxicity and metabolism of quinoxaline 1,4-di-N-oxides in vitro and in vivo. Drug Metab. Rev..

[44] Amić A., Lučić B., Stepanić V., Marković Z., Marković S., Dimitrić Marković J.M., Amić D. (2017). Free radical scavenging potency of quercetin catecholic colonic metabolites: Thermodynamics of 2H(+)/2e(-) processes. Food Chem..

[45] Dong B., Shi Z., Dong Y., Chen J., Wu Z.X., Wu W., Chen Z.S., Han C. (2022). Quercetin ameliorates oxidative stress-induced cell apoptosis of seminal vesicles via activating Nrf2 in type 1 diabetic rats. Biomed. Pharm..

[46] Feng K., Chen Z., Pengcheng L., Zhang S., Wang X. (2019). Quercetin attenuates oxidative stress-induced apoptosis via SIRT1/AMPK-mediated inhibition of ER stress in rat chondrocytes and prevents the progression of osteoarthritis in a rat model. J. Cell Physiol..

[47] Ramyaa P., Padma V.V. (2013). Ochratoxin-induced toxicity, oxidative stress and apoptosis ameliorated by quercetin--modulation by Nrf2. Food Chem. Toxicol..

[48] Li C., Zhang W.J., Choi J., Frei B. (2016). Quercetin affects glutathione levels and redox ratio in human aortic endothelial cells not through oxidation but formation and cellular export of quercetin-glutathione conjugates and upregulation of glutamate-cysteine ligase. Redox Biol..

[49] Nna V.U., Usman U.Z., Ofutet E.O., Owu D.U. (2017). Quercetin exerts preventive, ameliorative and prophylactic effects on cadmium chloride—Induced oxidative stress in the uterus and ovaries of female Wistar rats. Food Chem. Toxicol..

[50] Chen J.Y., Hu R.Y., Chou H.C. (2013). Quercetin-induced cardioprotection against doxorubicin cytotoxicity. J. Biomed. Sci..

[51] Weinberg S.E., Chandel N.S. (2015). Targeting mitochondria metabolism for cancer therapy. Nat. Chem. Biol..

[52] Brand M.D., Affourtit C., Esteves T.C., Green K., Lambert A.J., Miwa S., Pakay J.L., Parker N. (2004). Mitochondrial superoxide: Production, biological effects, and activation of uncoupling proteins. Free Radic. Biol. Med..

[53] Wang X., Yang P., Li J., Ihsan A., Liu Q., Cheng G., Tao Y., Liu Z., Yuan Z. (2016). Genotoxic risk of quinocetone and its possible mechanism in in vitro studies. Toxicol. Res..

[54] Dai C., Li D., Gong L., Xiao X., Tang S. (2016). Curcumin Ameliorates Furazolidone-Induced DNA Damage and Apoptosis in Human Hepatocyte L02 Cells by Inhibiting ROS Production and Mitochondrial Pathway. Molecules.

[55] Dai C., Li J., Tang S., Li J., Xiao X. (2014). Colistin-induced nephrotoxicity in mice involves the mitochondrial, death receptor, and endoplasmic reticulum pathways. Antimicrob. Agents Chemother..

[56] Wang H., Liu H., Zheng Z.M., Zhang K.B., Wang T.P., Sribastav S.S., Liu W.S., Liu T. (2011). Role of death receptor, mitochondrial and endoplasmic reticulum pathways in different stages of degenerative human lumbar disc. Apoptosis.

[57] Dai C., Tang S., Deng S., Zhang S., Zhou Y., Velkov T., Li J., Xiao X. (2015). Lycopene attenuates colistin-induced nephrotoxicity in mice via activation of the Nrf2/HO-1 pathway. Antimicrob. Agents Chemother..

[58] Ojo O.O., Olorunsogo O.O. (2021). Quercetin and vitamin E attenuate diabetes-induced testicular anomaly in Wistar rats via the mitochondrial-mediated apoptotic pathway. Andrologia.

[59] Fang P., Liang J., Jiang X., Fang X., Wu M., Wei X., Yang W., Hou W., Zhang Q. (2020). Quercetin Attenuates d-GaLN-Induced L02 Cell Damage by Suppressing Oxidative Stress and Mitochondrial Apoptosis via Inhibition of HMGB1. Front. Pharmacol..

[60] Chakraborty J., Pakrashi S., Sarbajna A., Dutta M., Bandyopadhyay J. (2022). Quercetin Attenuates Copper-Induced Apoptotic Cell Death and Endoplasmic Reticulum Stress in SH-SY5Y Cells by Autophagic Modulation. Biol. Trace Elem. Res..

[61] Zhang D.D., Chapman E. (2020). The role of natural products in revealing NRF2 function. Nat. Prod. Rep..

[62] Shinkai Y., Kimura T., Itagaki A., Yamamoto C., Taguchi K., Yamamoto M., Kumagai Y., Kaji T. (2016). Partial contribution of the Keap1-Nrf2 system to cadmium-mediated metallothionein expression in vascular endothelial cells. Toxicol. Appl. Pharm..

[63] Granado-Serrano A.B., Martín M.A., Bravo L., Goya L., Ramos S. (2012). Quercetin modulates Nrf2 and glutathione-related defenses in HepG2 cells: Involvement of p38. Chem. Biol. Interact..

[64] Yao P., Nussler A., Liu L., Hao L., Song F., Schirmeier A., Nussler N. (2007). Quercetin protects human hepatocytes from ethanol-derived oxidative stress by inducing heme oxygenase-1 via the MAPK/Nrf2 pathways. J. Hepatol..

[65] Li C., Zhang W.J., Frei B. (2016). Quercetin inhibits LPS-induced adhesion molecule expression and oxidant production in human aortic endothelial cells by p38-mediated Nrf2 activation and antioxidant enzyme induction. Redox Biol..

[66] Boots A.W., Drent M., de Boer V.C., Bast A., Haenen G.R. (2011). Quercetin reduces markers of oxidative stress and inflammation in sarcoidosis. Clin. Nutr..

[67] Nieman D.C., Henson D.A., Davis J.M., Angela Murphy E., Jenkins D.P., Gross S.J., Carmichael M.D., Quindry J.C., Dumke C.L., Utter A.C. (2007). Quercetin's influence on exercise-induced changes in plasma cytokines and muscle and leukocyte cytokine mRNA. J. Appl. Physiol..

[68] Lu N.T., Crespi C.M., Liu N.M., Vu J.Q., Ahmadieh Y., Wu S., Lin S., McClune A., Durazo F., Saab S. (2016). A Phase I Dose Escalation Study Demonstrates Quercetin Safety and Explores Potential for Bioflavonoid Antivirals in Patients with Chronic Hepatitis C. Phytother. Res. PTR.

