# Supplementary material for: Quercetin Attenuates Quinocetone-Induced Cell Apoptosis In Vitro by Activating the P38/Nrf2/HO-1 Pathway and Inhibiting the ROS/Mitochondrial Apoptotic Pathway
Source: Antioxidants (Basel). 2022 Jul 30;11(8):1498. doi: 10.3390/antiox11081498 (PMC9405464; doi:10.3390/antiox11081498)
Supplement: Supplementary file 1 [file antioxidants-11-01498-s001.zip › antioxidants-1806320-supplementary.pdf]

Suppl. Table 1. STR and amelogenin genotyping results of L02 cell line

Loci	Sample information			Cell Bank information		
	Sample name：1			Cell line name：L02 (CVCL_6926)		
	**Allele1**	**Allele2**	**Allele3**	**Allele1**	**Allele2**	**Allele3**
D5S818	11	12		11	12	
D13S317	13.3	13.3		13.3	13.3	
D7S820	12	12		12	12	
D16S539	9	10		9	10	
VWA	16	16		16	18	
TH01	7	7		7	7	
AMEL	X	X		X	X	
TPOX	8	12		8	12	
CSF1PO	10	10		10	10	
D12S391	20	20				
FGA	18	21				
D2S1338	17	17				
D21S11	27	28				
D18S51	16	16				
D8S1179	12	12				
D3S1358	15	18				
D6S1043	18	18				
PENTAE	7	17				
D19S433	13	13				
PENTAD	8	15				
D1S1656	12	15				

Suppl. Table 2. Primer sequences of the quantitative real-time PCR

Gene	Direction	Primer sequence (5′to 3′)
Nrf2	forward	5′- ACA CGG TCC ACA GCT CAT C -3′,
	reverse	5′- TGT CAA TCA AAT CCA TGT CCT G-3′
HO-1	forward	5′-ACA GGT TGA CAG AAG AGG CTA A-3′
	reverse	5′- AAC AGG AAG CTG AGA GTG AGG -3′
β-actin	Forward	5′- GCC GCC AGC TCA CCA TGG ATG -3′
	reverse	5′- GA CCC CGT CAC CGG AGT CCA -3′

Suppl. Figures:

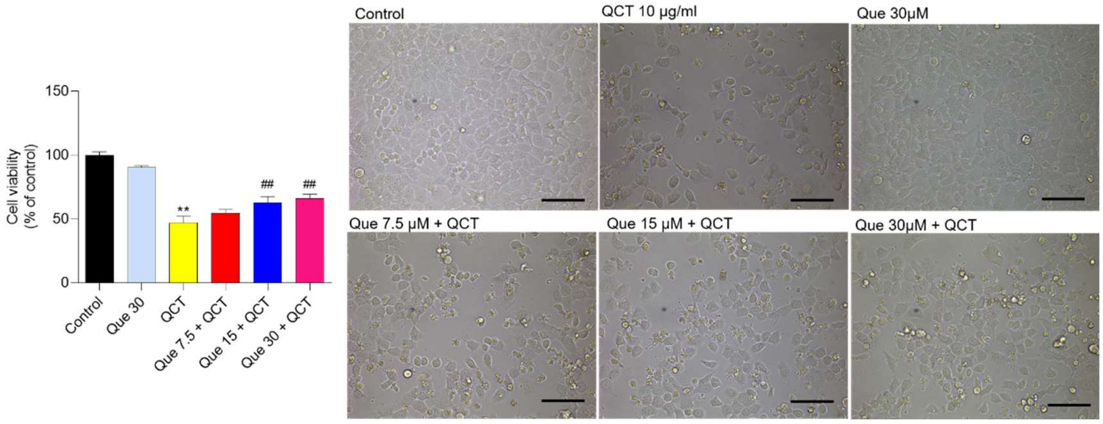

Supple. Figure S1. The effect of quercetin supplementation on QCT-induced cytotoxicity in HepG2 cells. HepG2 cells were pretreated with quercetin at the doses of 7.5, 15, and 30 μM , followed by treatment with QCT at the dose of 10 μg/mL for additional 24 h, then the cell viabilities (on the left) were measured and changes of cell morphology (on the right) were observed. All results were presented as mean ± SD (n =3 independent experiments). **P<0.01, compared to the vehicle control group; ##P<0.01, compared to the QCT alone group. QCT, quinocetone; Que, quercetin; Bar=50 μm.

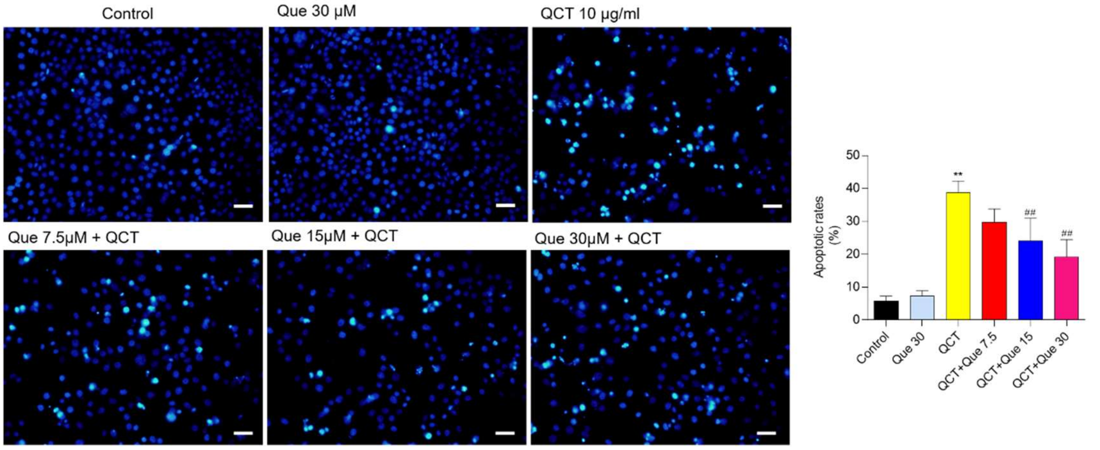

Supple. Figure S2. The effect of quercetin supplementation on QCT-induced cell apoptosis in HepG2 cells. HepG2 cells were treated with quercetin pretreatment at 7.5, 15, and 30 μM for 2 h, followed to co-treat QCT at the final concentration of 10 μg/mL for an additional 24 h, and cell apoptosis was stained with Hoechst 33342. Finally, the representative images were obtained using the fluorescence microscope (on the left) and apoptotic rates were quantified using Image J (on the right). All results were presented as mean ± SD (n = 3 independent experiments). **P<0.01, compared to the vehicle control group; ##P<0.01, compared to the QCT alone group. QCT, quinocetone; Que, quercetin; Bar = 50 μm.

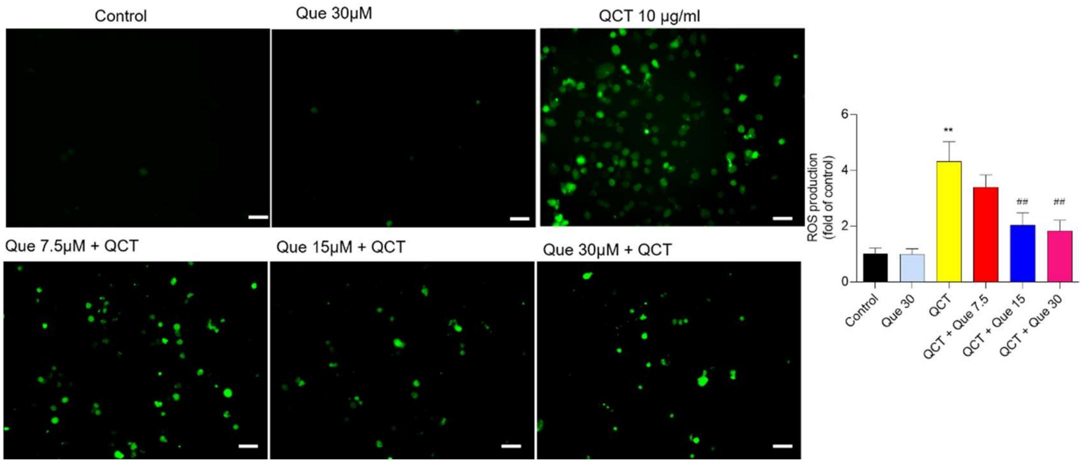

Supple. Figure S3. Effect of quercetin supplementation on QCT-induced ROS production and oxidative damage in human HepG2 cells. Intracellular ROS production was measured using the dye 2,7-dichlorofluorescein diacetate staining. The representative images were shown (on the left) and the fluorescence intensities were quantified using Image J (on the right). Bar = 50 μm. All results were presented as mean ± SD (n = 4 independent experiments). ***P*<0.01, compared to the vehicle control group; ##*P*<0.01, compared to the QCT alone group. QCT, quinocetone; Que, quercetin; Bar = 50 μm.

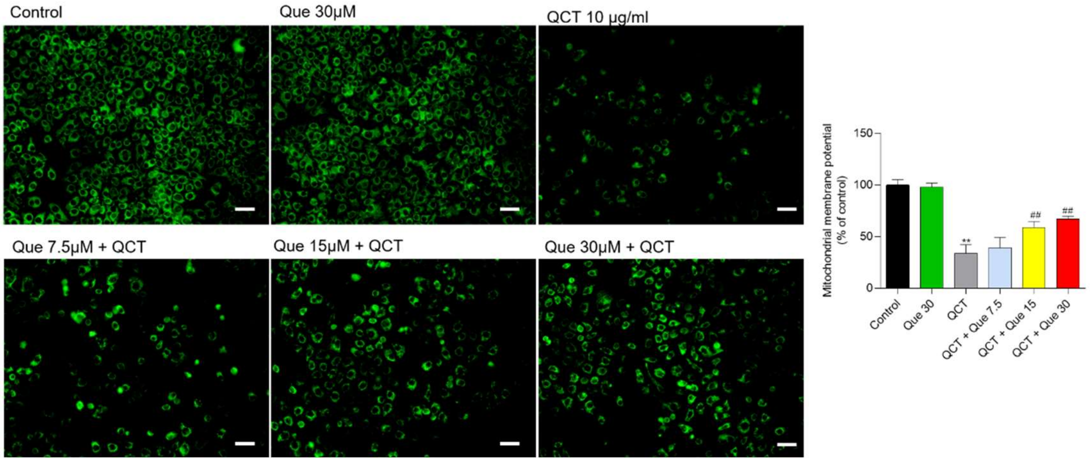

Supple. Figure S4. Effects of quercetin supplementation on QCT-induced loss of mitochondrial membrane potential (MMP) in HepG2 cells. The changes in mitochondrial membrane potential were examined by using rhodamine-123 staining. The representative images were shown (on the left) and the fluorescence intensities were quantified using Image J (on the right). Bar = 50 μm. All results were presented as mean ± SD (n = 4 independent experiments). ***P*<0.01, compared to the vehicle control group; ##*P*<0.01, compared to the QCT alone group. QCT, quinocetone; Que, quercetin; Bar = 50 μm.

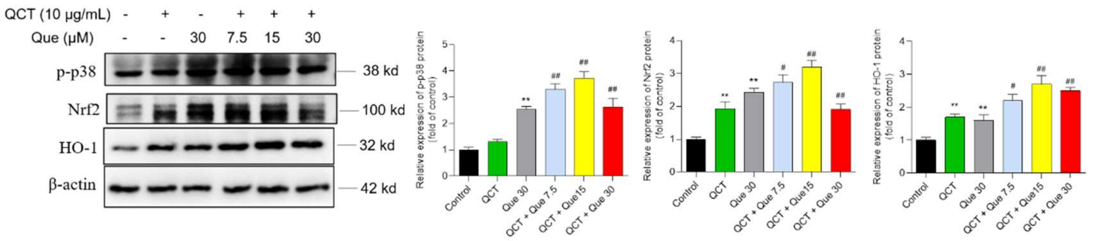

Supple. Figure S5. Quercetin supplementation upregulated the expression of Nrf2, HO-1, and p-p38 proteins in human HepG2 cells. HepG2 cells were treated with quercetin at the final concentrations of 7.5, 15, 30 μM for 2h, followed to treat with QCT at the dose of 10 μg/mL for additional 24 h. The expression of HO-1, Nrf2, and p-p38 expression were examined. The representative images were shown (on the left) and the values of each band were quantified using Image J (on the right). All results were presented as mean ± SD (n =3 independent experiments). $^{**}P<0.01$, compared to the vehicle control group; $^{\#\#}P<0.01$, compared to the QCT alone group. QCT, quinocetone; Que, quercetin.
